# Breast Conservation Surgery: State of the Art

**DOI:** 10.4061/2011/107981

**Published:** 2011-09-04

**Authors:** Jonathan White, Raj Achuthan, Philip Turton, Mark Lansdown

**Affiliations:** The Breast Care Unit, Leeds General Infirmary, Leeds, West Yorkshire LS1 3EX, UK

## Abstract

Breast conservation surgery is available to the vast majority of women with breast cancer. The combination of neoadjuvant therapies and oncoplastic surgical techniques allows even large tumours to be managed with a breast-conserving approach. The relationship between breast size and the volume of tissue to be excised determines the need for volume displacement or replacement. Such an approach can also be used in the management of carefully selected cases of multifocal or multicentric breast cancer. The role of novel techniques, such as endoscopic breast surgery and radiofrequency ablation, is yet to be precisely defined.

## 1. Introduction

The replacement of obligatory mastectomy, be it radical or modified radical, by simple mastectomy or wide local excision and adjuvant radiotherapy, reflected a paradigm shift in the understanding of breast cancer pathology and biology [1]. The combination of multimodal treatments, both locoregional, in the form of conservation surgery and radiotherapy, and systemic endocrine treatment and chemotherapy, has resulted in reduced postsurgical morbidity without compromising oncological outcomes [2]. The concept of downstaging tumours by means of neo-adjuvant chemotherapy or endocrine therapy is increasingly being applied to improve the chance of successful conservation surgery in the same way as it can render operable the inoperable [3, 4]. The adoption of oncoplastic surgical techniques allows larger tumours to be excised safely without compromising cosmetic outcomes. Currently, the only absolute contraindications to breast conservation relate to tumours with chest wall involvement, significant skin involvement, and patients with either extensive malignant microcalcifications or inflammatory carcinoma [5, 6]. Multifocal and multicentric tumours remain relative contraindications to attempts at breast conserving surgery. Such patients need careful counselling regarding the possible need for further surgery if excision is incomplete, and the increased risk of locoregional recurrence. Meticulous preoperative planning is essential if conservation is to be successful in this context [7]. The role of salvage breast conservation surgery in the management of local recurrence, or a metachronous primary cancer, is controversial, and should be considered with caution.

While some studies in the United States have suggested that the increasing incidence of breast cancer may have begun to level off [8], data from European countries does not reflect this change [9, 10]. Survival from breast cancer has certainly improved [11, 12], and it follows, therefore, that there are more patients alive now, having survived breast cancer, than at any other time. Aesthetic concerns and expectations are understandably higher on patients' agenda than previously and remain a source of psychological morbidity after mastectomy or if the results from breast conservation surgery are poor [12, 13]. Thus the importance of the oncoplastic approach, defined as the application of plastic surgery techniques of partial breast reconstruction at the time of breast cancer surgery, to optimising the oncological and cosmetic outcomes of breast conservation, has never been more keenly felt [14–16].

## 2. Optimisation of Oncological Factors

Tumours may be successfully downstaged with neoadjuvant chemotherapy and/or endocrine therapy, allowing the majority of patients to undergo breast conservation surgery [4, 17]. In this context, the decision to proceed with breast conserving surgery is guided by the clinical and radiological response to neoadjuvant therapy. Magnetic resonance imaging is superior to mammography or ultrasound in evaluating the response to neoadjuvant therapy and should be used in preference [18–20]. Whereas regimens of neoadjuvant chemotherapy usually last approximately six months, the duration of endocrine therapy in the neoadjuvant context is more varied. Although only used for three months in the IMPACT trial [21], reductions in tumour size were sufficient to allow breast conservation treatment in a large proportion. Neoadjuvant endocrine treatment is sometimes associated with a more gradual reduction in tumour size and can safely be continued for longer durations prior to undertaking curative breast conservation surgery [22]. 

Small lesions, which are impalpable or difficult to feel, should be localised stereotactically or by ultrasound. A number of techniques are available, utilising hookwire localisation, radioactive beads, or injection of radioisotope colloid, the latter being particularly attractive in cases where breast-conserving surgery is performed in conjunction with sentinel lymph node biopsy [23, 24].

The use of intraoperative specimen X-ray helps confirm complete excision of the radiological abnormality [25]. This has been shown to help reduce the need for further surgery because of margin positivity, as a further cavity shave may be taken intraoperatively if the specimen X-ray gives cause for concern [26].

Optimal oncological treatment demands complete excision of malignant tissues with a negative resection margin. What constitutes a negative margin is not well defined. In early studies, only margins of >1 cm were considered negative [27, 28]. A recent meta-analysis showed equivalent rates of local recurrence with margins as close as 1-2 mm [29, 30], but closer margins have been associated with rates of local recurrence similar to those seen in cases with positive margins [31, 32].

The use of intraoperative frozen section for assessment of margins, where available, is helpful in reducing the number of second procedures required to achieve clear margins [33, 34]. A further cavity shave can be taken from any margin found to be positive on intraoperative frozen section. Intraoperative touch imprint cytology can also be used as a means of margin assessment but, as with frozen section, requires the availability of an expert cytopathologist to report slides intraoperatively [35]. 

The role of routine cavity biopsies is controversial [36]. Hewes et al. (2009) found poor correlation between the status of the resection margin and cavity biopsies. In their series, the status of cavity biopsies was a better predictor of both breast-cancer-specific and overall survival. The two key benefits of this approach are the reduced need for second operations if the specimen margin is positive and the cavity biopsy negative and the diagnosis of otherwise occult multifocal disease, often necessitating mastectomy [37]. Conversely, it can be argued that the practice is both unnecessary, given that discontinuous small foci of disease are adequately treated by radiotherapy [28] and undesirable, as it inevitably results in the excision of more tissue than strictly necessary, having a potentially adverse effect on cosmetic outcomes.

Rates of local recurrence after breast-conserving surgery are significantly reduced by the use of adjuvant radiotherapy, giving rates of overall survival similar to those following mastectomy [38], and therefore should be viewed as a standard of care, unless distant metastases are discovered soon after surgery [39, 40]. Postoperative external beam whole-breast radiotherapy remains the most commonly used technique, although partial breast radiotherapy is possible, and may be performed intraoperatively or postoperatively, via external beam, brachytherapy, or photon emission [41–44]. There is evidence that partial breast radiotherapy may be superior in terms of cosmetic outcome [45]. This is often cited as a major advantage over whole breast radiotherapy, which is associated with a number of unfavourable cosmetic sequelae, such as breast lymphoedema, fibrosis, and shrinkage of the breast tissue, often leading to accentuation of small parenchymal defects and distortion of the nipple. However, although short-term rates of local recurrence after partial breast irradiation seem similar, long-term data (i.e., over 10 years) showing equivalence with traditional whole-breast external beam radiotherapy are not yet available. The importance of reducing rates of 5-year locoregional recurrence is emphasised by its relationship with 15-year mortality. The 20% reduction in 5-year locoregional recurrence associated with the addition of radiotherapy to breast conservation surgery corresponds to a 5% reduction in mortality at 15 years [46].

## 3. Optimisation of the Cosmetic Outcome after Breast Conservation Surgery

The cosmetic appearance of the breast after breast conservation surgery depends, firstly, on the relative proportion of breast volume excised in order to satisfy oncological requirements and, secondly, on the location of the tumour within the breast.

The cosmetic defect caused by excision of medial tumours, especially in the upper inner quadrant, is more pronounced than for tumours in the outer half of the breast. Estimation of the proportion of breast volume to be excised is therefore an important consideration when planning surgery [47]. Successful oncological and aesthetic outcome depends on adequate preoperative planning. Mammography and ultrasound alone may underestimate the extent of disease and fail to demonstrate multifocality. Magnetic resonance imaging is being used increasingly in this context, as it has been shown to give a more accurate estimate of the true distribution of malignancy, particularly for lobular carcinomas [48, 49].

## 4. Surgical Principles

*Overview*.

- General principles
  - choice of incision,
  - avoidance of nipple deviation.
- Techniques for excision of <10% of breast volume.
- Techniques for excision of 10–20% breast volume:
  - volume displacement,
  - central tumours,
  - peripheral tumours.
- Techniques for excision of >20% of breast volume:
  - tissue transfer.

Incisions should follow Langer's lines, semicircular, concentric to the edge of the areola, or Kraissl's lines, parallel to the horizontal skin creases. Radial incisions can be useful, but care must be taken to ensure that the nipple-areola complex is not likely to be displaced as the scar contracts during the process of wound healing and radiotherapy. A circumareolar incision can give good access to most lesions except those at the extreme periphery of the breast.

The skin overlying the cancer only needs to be excised if there are concerns regarding skin involvement, for example, if there is in-drawing of the skin or fixed dimpling. Following wide excisions, the resultant scarring and radiotherapy changes tend to cause nipple deviation towards the scar. This can be avoided by undermining the skin and disconnecting the ducts behind the nipple-areola complex. If needed, the remaining glandular tissue can also be undermined to allow rotation and approximation of tissue into the defect. If significant NAC deviation is anticipated, then de-epithelialisation of a crescent of skin from the areolar edge that is opposite to the scar and resiting the nipple to adjust for anticipated deviation often is helpful.

**Figure 1:**
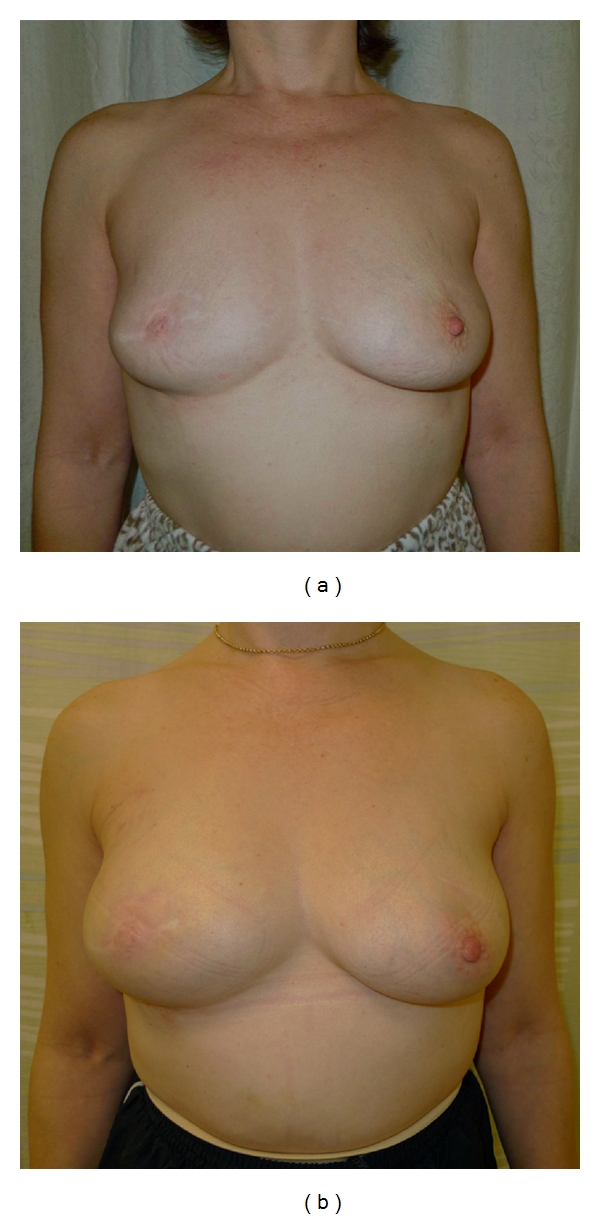
(a) This patient had previously undergone a central wide local excision and nipple reconstruction at the age of 47. Although the contour of the right breast is similar to that of the left, there is a relative lack of projection and the breast has a blunted appearance. (b) Autologous fat transfer, in the form of lipomodelling, successfully fills the defect from previous surgery. The patient also received a subdermal silicone areola prosthesis to improve projection of the reconstructed nipple.

In general, excision of up to 10% of breast volume as a simple wide local excision gives an acceptable cosmetic result [47]. The resultant filling defect can be resolved to some degree by generously undermining the surrounding glandular tissue to allow it to fill the wide excision cavity. For those cases with defects despite breast remodelling the use of autologous fat transfer (Figures 1(a) and 1(b)) is emerging as an attractive option [50]. However, dystrophic calcification following fat necrosis may result in increased recall after screening mammography for biopsy [51]. 

For cancers occupying up to 20% of breast volume, some degree of volume displacement may be required to fill the defect [52]. This is achieved by mobilisation and transposition of neighbouring glandular tissue with or without overlying skin (see Table 1). Suitable patients with adequate breast volume may wish to undergo therapeutic mammoplasty [53]. Surgery to the contralateral breast may be requested to improve symmetry and may take the form of a reduction mammoplasty or mastopexy.

For cancers occupying 20–40% of the breast, volume displacement alone may not be sufficient and thus volume replacement by autologous tissue transfer may become necessary.

## 5. Optimising Cosmesis: Central Tumours Occupying 10–20% of Breast Volume

Subareolar tumours have previously been viewed as an indication for mastectomy but may be safely approached by central excision with resection of the nipple-areola complex [54, 55]. The skin wound can be closed with a purse string or horizontal suture, although this tends to reduce the projection of the breast mound. A central excision with volume displacement using a Grisotti dermoglandular flap is more appropriate for larger breasts with greater degrees of ptosis [56]. After excision of the nipple-areola complex and the underlying tumour, a dermoglandular flap is harvested from the inferolateral breast. The flap is then de-epithelialised apart from the circle of skin destined to reconstruct the nipple. Free rotation depends on the flap being freed from the prepectoral fascia. An inverted-T (WISE pattern) mammoplasty [53], excising the nipple-areola complex, is a popular alternative, with the nipple potentially being reconstructed at a later date.

**Figure 2:**
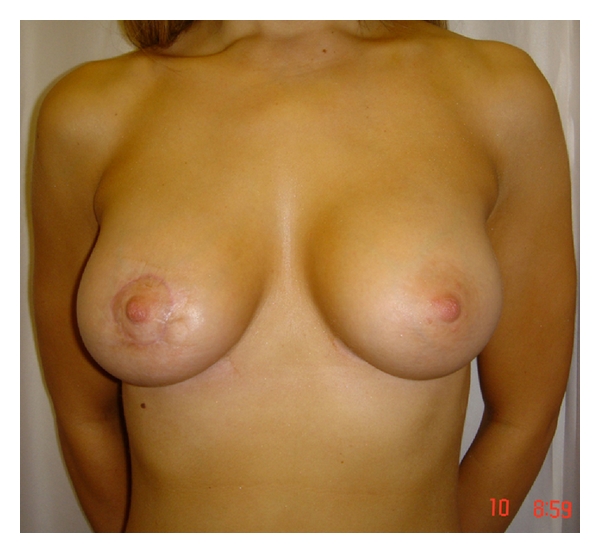
This patient presented with a 1 cm tumour located in the upper inner quadrant of the right breast. The tumour was excised via a periareolar incision and the remaining breast tissue was mobilised to close the defect. The round block technique ensured that the nipple-areolar complex remained in the correct position.

For central tumours not involving the nipple-areola complex an alternate option would be the use of Benelli's round block technique (Figure 2) [57]. Concentric circles are incised around the areola, and the skin resected, allowing access to the periareolar tissue. This allows reshaping of the breast by mobilising adjacent tissue, and the skin is closed by means of a purse string suture [58]. Alternatively such tumours may be excised in combination with a batwing mastopexy, otherwise known as the omega plasty, while preserving the nipple-areola complex [59]. Briefly, semicircular incisions are made: one circumareolar and the other a short distance away, and these are joined by angled “wings” to each side of the areola. After excision of the breast lesion, the defect is closed by advancing the breast tissue and closing the skin.

## 6. Optimising Cosmesis: Peripheral Tumours Occupying 10–20% of Breast Volume

Different oncoplastic techniques lend themselves to excision of lesions in certain locations (see Table 2). Tumours above the nipple-areola complex may be excised and the defect filled with an inferior pedicle mammoplasty. 

**Figure 3:**
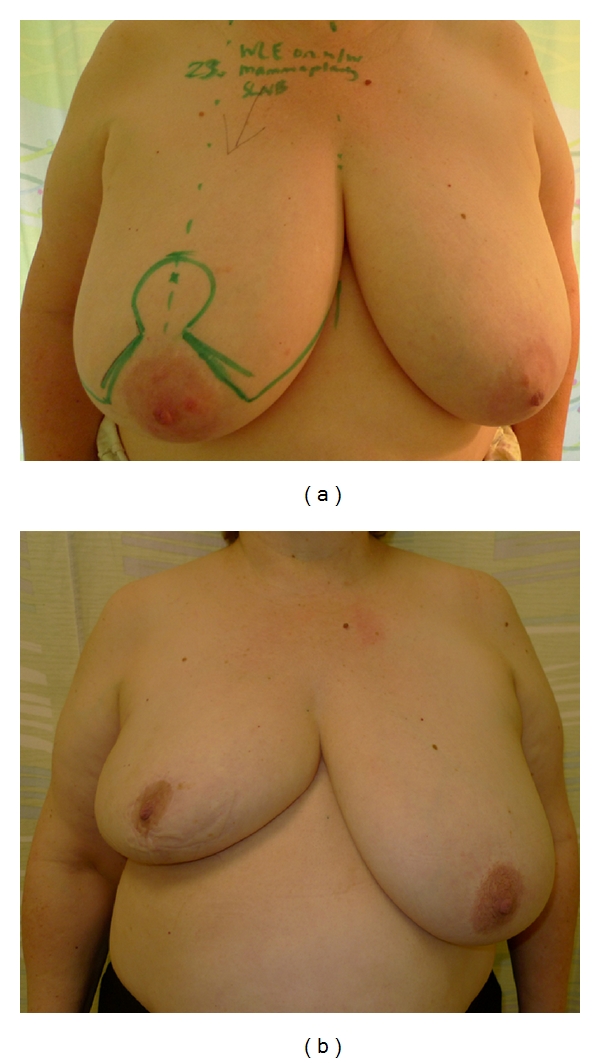
(a) This 63-year-old patient with large ptotic breasts presented with a tumour in the right breast. The skin markings show the planned incisions for a therapeutic inverted-T mammoplasty. (b) Postoperative images of the same patient after completion of adjuvant chemotherapy and prior to commencing radiotherapy. The inverted-T mammoplasty gives a satisfactory result and is in proportion to the patient's body habitus. Reduction mammoplasty of the contralateral breast, to improve symmetry, is planned to be performed six months after completion of adjuvant radiotherapy.

Excision of tumours in the lateral aspect of the breast: tumours inferior to the nipple-areola complex may be excised by means of a vertical mammoplasty [60, 61] or nipple-sparing inverted-T mammoplasty [53] (Figures 3(a) and 3(b)). Moderately sized tumours in the lower outer quadrant may be resected using a modified approach, sometimes referred to as the J-mammoplasty, with larger tumours excised via an inverted-T or L-mammoplasty.

Tumours close to the inframammary fold may be removed by excising an ellipse of skin and breast tissue and simply closing the resulting defect. Although this reduces the distance from nipple to inframammary fold, this is often not apparent in patients with preexisting ptosis.

## 7. Optimising Cosmesis after Extensive Excision of 20–40% of Breast Volume: Techniques of Tissue Transfer

**Figure 4:**
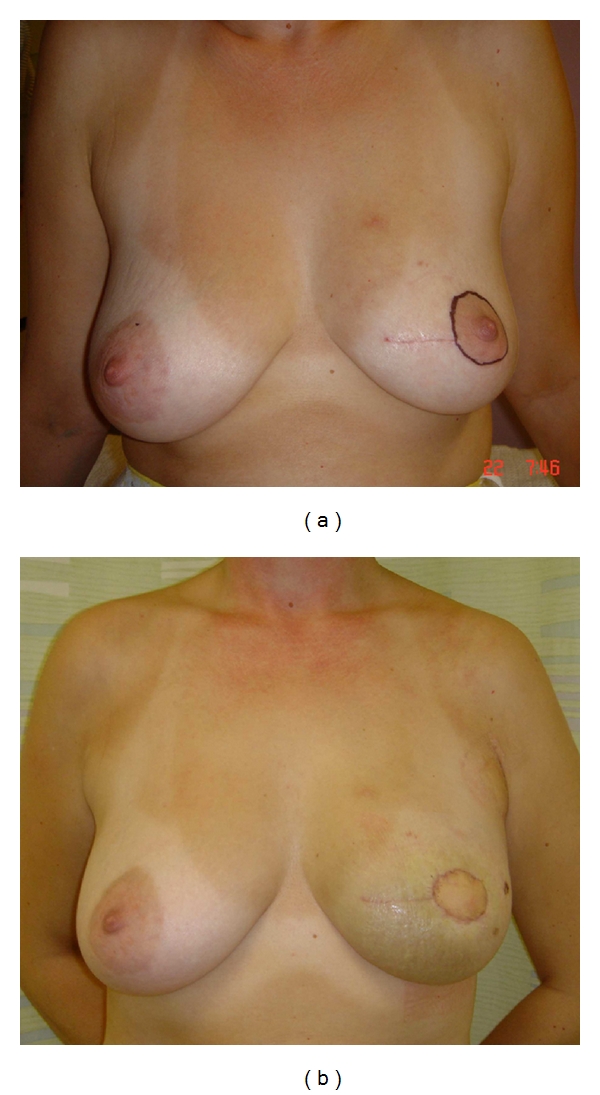
(a) This 41-year-old patient had previously undergone wide local excision of a tumour in the left breast. The lateral margin was involved, necessitating a further central wide local excision to include the nipple-areolar complex. (b) In view of the predicted loss of volume, a *latissimus dorsi* miniflap was utilised to both fill the resultant defect and also replace the skin of the areola. The volume of the partially reconstructed breast is very similar to that of the contralateral side, although postoperative swelling is apparent.

**Figure 5:**
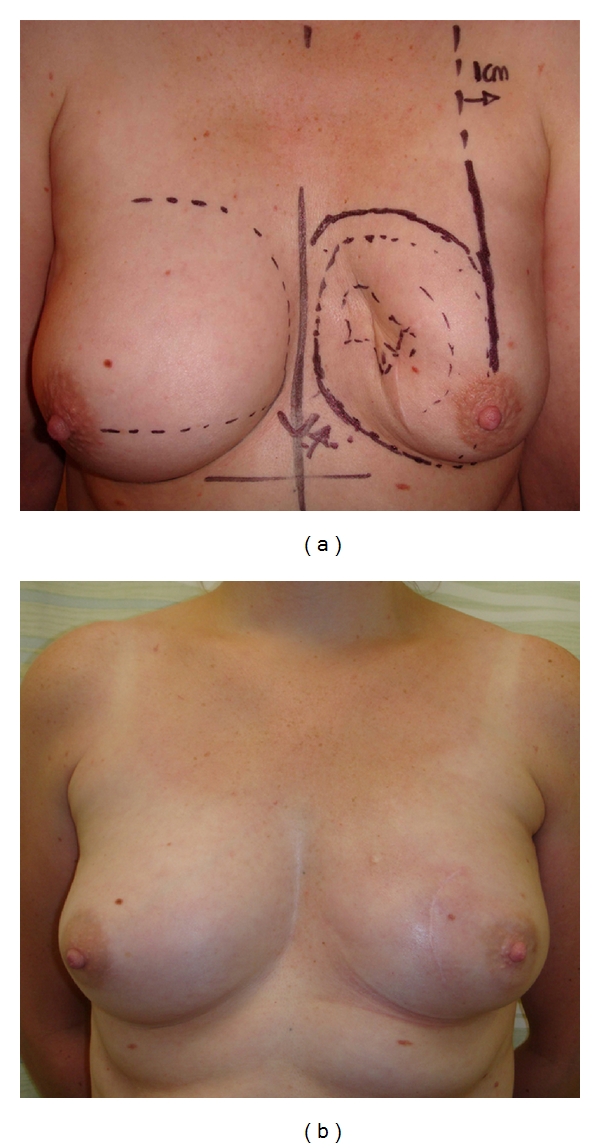
(a) This patient had previously undergone wide local excision and adjuvant radiotherapy for a cancer located in the lower inner quadrant of the left breast. The resulting defect causes significant distortion to the breast shape and nipple deviation toward the midline. (b) An omental flap was harvested laparoscopically in order to partially reconstruct the breast, achieving a high degree of symmetry with the contralateral breast.

When more than 20% of breast volume is excised, tissue mobilisation alone may not succeed in achieving a satisfactory result and, unless the patient desires a much smaller breast, volume replacement by tissue transfer may be necessary. Most commonly, this entails use of a pedicled *latissimus dorsi* miniflap (Figures 4(a) and 4(b)), which can be mobilised to fill a defect in any quadrant [62, 63]. The first stage of the procedure involves excision of the breast lesion, and then the latissimus dorsi miniflap is used to fill the defect after a delay of one or two weeks to allow the margin status to be assessed [64]. If intraoperative analysis of surgical margins by frozen section is available, then a single stage procedure is feasible [65, 66]. Alternatives include mobilisation of axillary tissue on a thoracodorsal artery perforator lipodermal flap [7] or use of intercostal artery perforator flaps [66]. One novel approach adopted in our unit is to laparoscopically harvest an omental flap (Figures 5(a) and 5(b)) to fill the local defect [67]. Whereas pedicled flaps usually withstand radiotherapy, albeit with a substantial rate of complications, the use of free flaps in this context is contraindicated.

## 8. Optimising Management of Multifocal and Multicentric Tumours

The management of multifocal tumours, within the same quadrant, and multicentric tumours, in different quadrants or in the same quadrant but widely separated (>5 cm), is controversial. Traditionally these scenarios would dictate mastectomy as the only oncologically sound procedure. If a conservative procedure is to be considered in these patients, careful selection is required with regard to tumour location and breast size and shape and counselled regarding the increased likelihood of further surgery should margins be positive, and possible increased risk of local recurrence, which may entail completion mastectomy. There have been no randomised controlled trials to address the issue of the oncological safety of breast conservation surgery in this context [7]. A retrospective study comparing outcomes of patients with multifocal and unifocal cancers showed equivalent overall survival and no increase in risk of locoregional recurrence [68].

In general, tumours closely spaced within the breast may be removed together utilising an appropriate technique such as an omegaplasty or inverted-T mammoplasty as listed above, whereas separate wide excisions are more appropriate for tumours separated by >5 cm. Careful preoperative planning is of paramount importance. This may often include the use of magnetic resonance imaging, and image-guided localisation of all lesions to be excised is essential. Access to intraoperative frozen section histology, while desirable in terms of reducing the need for further surgery in case of margin involvement, is not an absolute prerequisite.

## 9. Optimising Management of Local Recurrence or Metachronous Ipsilateral Primary Breast Cancer

The role of further attempts at breast conservation in patients who have previously undergone wide local excision for an ipsilateral cancer is controversial. Whole-breast radiotherapy can only be given once, and therefore further breast conservation surgery alone, versus mastectomy, is subject to the same disparity in efficacy as when wide local excision, without radiotherapy, is compared with mastectomy for primary breast cancer [28]. Thus, perhaps as many as 40% of women treated in this way will have further problems with local recurrence. Given these odds, many women will opt for mastectomy rather than any further attempt at breast conservation, but partial breast radiotherapy may be used in this context in an attempt to reduce the risk of failure [69]. At present, partial breast radiotherapy is only offered to a minority of patients. As these techniques gain wider acceptance and enter routine practice, a greater proportion of patients may be eligible for further breast conservation surgery to manage local recurrence or metachronous ipsilateral primary breast cancer.

## 10. Optimising Symmetry: When to Perform Contralateral Surgery

**Figure 6:**
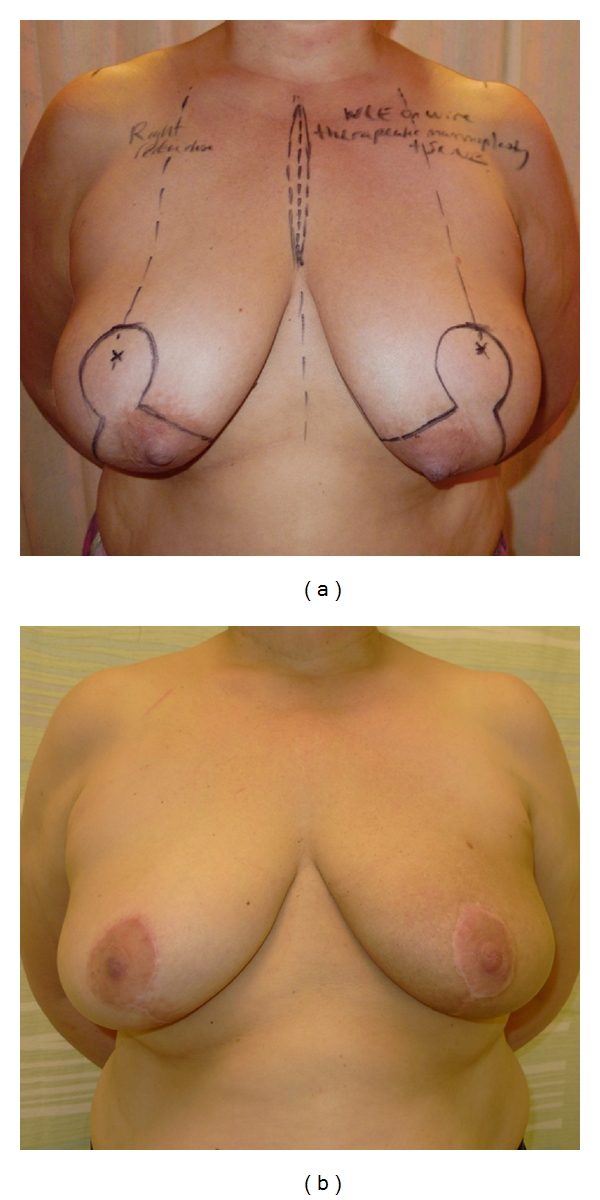
(a) This 51-year-old patient with large, ptotic breasts and nipple-areola complexes situated medial to the breast meridian presented with a left breast cancer. These images show the skin markings used to plan a therapeutic inverted-T mammoplasty and simultaneous contralateral reduction mammoplasty for symmetrisation. (b) Adjuvant radiotherapy to the left breast has resulted in mild changes in skin pigmentation but symmetry is still good with the contralateral breast being still satisfactory.

Large volume excisions, in patients for whom breast reduction is desirable, often result in noticeable asymmetry, which should be corrected. There is no consensus regarding the optimal timing of contralateral surgery. Simultaneous procedures are attractive in terms of reducing patient inconvenience and the need for a second admission and general anaesthetic (Figures 6(a) and 6(b)). Conversely, postradiotherapy changes can be unpredictable, and, therefore, some prefer to perform the contralateral reduction after these have had time to settle, to improve the chance of achieving good symmetry. A delayed approach also takes into account the possibility that further surgery may be required in the form of excision of margins or completion mastectomy if excision is incomplete [59]. If contralateral reduction is planned as a simultaneous procedure, then slightly more tissue should be excised and the nipple placed marginally higher, to mimic the predicted postradiotherapy shape [55].

## 11. Novel Technologies and the Future of Optimising Breast Conservation

Endoscopic breast surgery for benign and malignant disease has been described in a number of small case series [70, 71]. Carbon dioxide insufflation creates a working space and both subcutaneous mastectomy and wide local excision have been performed using this technique. Although usually employed in the management of ductal carcinoma *in situ*, excision of T1 carcinomas has also been successfully performed [72]. The ability to reliably excise tumours with clear surgical margins is not well established due to the small size of these case reports, and more work is needed before they will be readily adopted into routine practice [73].

Radiofrequency ablation for small breast tumours is currently under evaluation [74]. The procedure can be monitored intraoperatively by ultrasound and postoperatively by magnetic resonance imaging. Wide local excision may be performed after radiofrequency ablation to ensure adequate oncological treatment [75]. Concerns regarding the ability to accurately assess response by magnetic resonance imaging alone currently preclude the use of this technique in isolation [76]. Fine-needle aspiration cytology in conjunction with magnetic resonance imaging has been used to assess response in patients not undergoing excision [77], but this approach should not be employed outside of clinical research given its unproven sensitivity and inability to adequately sample the “margin” of ablation and because of the paucity of data related to long-term outcomes.

## 12. Conclusion

The role of surgery in the management of breast cancer has changed markedly since the days of Halsted, reflecting the change in the way breast cancer is perceived as a systemic, rather than locoregional, disease process. Multimodal therapies, especially in the form of neoadjuvant chemotherapy and endocrine therapy, have increased the proportion of women eligible for breast conservation. The adoption of relatively straightforward surgical techniques to achieve volume displacement can give superior cosmetic outcomes for patients with larger tumours. Techniques of volume replacement are more demanding but are within the remit of surgeons with an interest in oncoplastic surgery or can be performed in conjunction with a plastic surgeon.

The management of multifocal or multicentric cancers and the management of further conservation surgery for recurrence or metachronous ipsilateral primary after previous wide local excision are contentious issues. Ideally, multicentre randomised controlled trials should be designed to address these issues. Surgery to the contralateral breast to improve symmetry should be offered to all patients. The timing of such surgery, and the merits of synchronous versus delayed approaches, should be discussed with patients in full.

In the future, endoscopic breast cancer surgery and radiofrequency ablation therapy are likely to become more popular, but larger studies with longer periods of followup are needed to evaluate their oncological safety prior to their widespread adoption.

Now that patients are benefiting from improved disease-free and overall survival, the cosmetic outcome is of great importance as patients seek to come to terms with the aftermath of breast cancer and its treatment. The importance of cosmesis, in terms of emotional and psychosexual well-being [78–80], demands that the principles of an oncoplastic approach to breast conservation surgery be employed in treating all women with breast cancer.

## Figures and Tables

**Table 1:** 

Mammoplasty techniques for resection of 10–20% of breast volume:	
Glandular remodelling	
Inferior pedicle	
Superior pedicle	
Vertical scar	
Round block	
Grisotti flaps	

**Table 2:** Oncoplastic techniques suitable for excision of lesions in specified locations.

Tumour location	Oncoplastic technique
Superior to NAC	Periareolar (Benelli) mammoplasty
	Inferior pedicle (Grisotti) mammoplasty
Lateral to NAC	Lateral mammoplasty
Medial to NAC	Medial mammoplasty
Lower outer/inner quadrant	L-mammoplasty
	J-mammoplasty
Inferior to NAC	Vertical scar mammoplasty
	Inverted T (WISE) mammoplasty
Inframammary fold	IMF-plasty
